# Predictors of Acute Vertebrobasilar Vasospasm following Tumor Resection in the Foramen Magnum Region

**DOI:** 10.1371/journal.pone.0163908

**Published:** 2016-09-28

**Authors:** Chuanyuan Tao, Jiajing Wang, Yuekang Zhang, Shirong Qi, Fan Liu, Chao You

**Affiliations:** 1 Department of Neurosurgery, West China Hospital, Sichuan University, Chengdu, China; 2 Department of Critical Care Medicine, Neurosurgical Intensive Care Unit, West China Hospital, Sichuan University, Chengdu, China; Universitatsklinikum Freiburg, GERMANY

## Abstract

**Objective:**

Cerebral vasospasm can occur after skull base tumor removal. Few studies concentrated on the posterior circulation vasospasm after tumor resection in the posterior fossa. We aimed to identify the risk factors associated with postoperative vertebrobasilar vasospasm after tumor resection in the foramen magnum.

**Methods:**

We retrospectively reviewed the data of 62 patients with tumors in the foramen magnum at our institution from January 2010 to January 2015. The demographic data, tumor features, surgical characteristics were collected. Vertebrobasilar vasospasm was evaluated by bedside transcranial Doppler before surgery and on postoperative day 1, 3, 7. Univariate and multivariate analyses were performed to determine the predictors of postoperative vasospasm in the posterior circulation.

**Results:**

Vertebrobasilar vasospasm was detected in 28 (53.8%) of the 62 patients at a mean time of 3.5 days after surgery. There were 5 (8%) patients with severe vasospasm according to the grading criteria. Age, tumor type, tumor size, vertebral artery encasement, and surgical time were significantly related to vasospasm in the univariate analysis. Further multivariate analysis demonstrated that only age and vertebral artery encasement were independent risk factors predicting the occurrence of postoperative vertebrobasilar vasospasm.

**Conclusions:**

The incidence of acute vertebrobasilar vasospasm is not uncommon after foramen magnum tumor resection. Age and vertebral artery encasement are significantly correlated with postoperative vasospasm. Close monitoring of vasospasm should be given to patients with younger age and the presence of vertebral artery encasement on the preoperative imaging to facilitate early diagnosis and intervention.

## Introduction

Cerebral vasospasm is a common sequela after subarachnoid hemorrhage due to an aneurysmal rupture or neurotrauma[1, 2]. It can also occur following brain tumor resection, especially after skull base tumor removal[3–7]. Patients with middle fossa tumors are prone to suffering cerebral vasospasm as these tumors are in close contact with arteries in the circle of Willis[3, 5]. Tumors of the posterior fossa associated with postoperative vasospasm were relatively rare and their removal more usually involved arteries in the posterior circulation[5]. Recently, however, more than half of acoustic neuromas removal were reported to produce cerebral vasospasm[6]. This implies that the occurrence of vasospasm after tumor resection in the posterior fossa may be underestimated previously[6].

Foramen magnum (FM) is a typical area in the posterior fossa where tumors frequently have an intimate relationship with vertebral artery and sometimes basilar artery as well[8]. Vertebrobasilar artery can be displaced, narrowed or even encased by tumors in the FM[9, 10]. Moreover, mechanical manipulation of the vertebrobasilar artery may go through the whole surgical procedure[11, 12]. Therefore, resection of tumors in the FM poses a high risk of postoperative vertebrobasilar vasospasm theoretically and FM is an excellent region where the effect of posterior fossa tumor removal on vertebrobasilar vasospasm can be investigated. However, to the best of our knowledge, no study concentrated on postoperative vertebrobasilar vasospasm in this location.

Noninvasive, portable, and radiation-free transcranial Doppler (TCD) ultrasonography has been validated against angiography to detect vasospasm in the anterior circulation[13, 14], but its application in the posterior circulation remains controversial because of technical challenge and parameters of lower accuracy [15, 16]. Until recently, Soustiel et al. evaluated the value of an basilar artery/ extracranial vertebral artery (BA/EVA) flow velocity (FV) ratio combined with BA velocity to cope with the shortcoming of TCD. They showed that BA/EVA ratio, also named Soustiel’s ratio, could enhance the accuracy and reliability of TCD in the diagnosis of BA vasospasm[17]. Another study by Sviri et al. further verified the accuracy of the Soustiel’s ratio[18].

The present study aimed to evaluate the incidence and the risk factors of acute vertebrobasilar vasospasm by TCD after surgical resection of tumors in the FM, which may facilitate the early diagnosis and timely intervention and finally improve the surgical outcome.

## Material and Methods

### Patients

**Fig 1:**
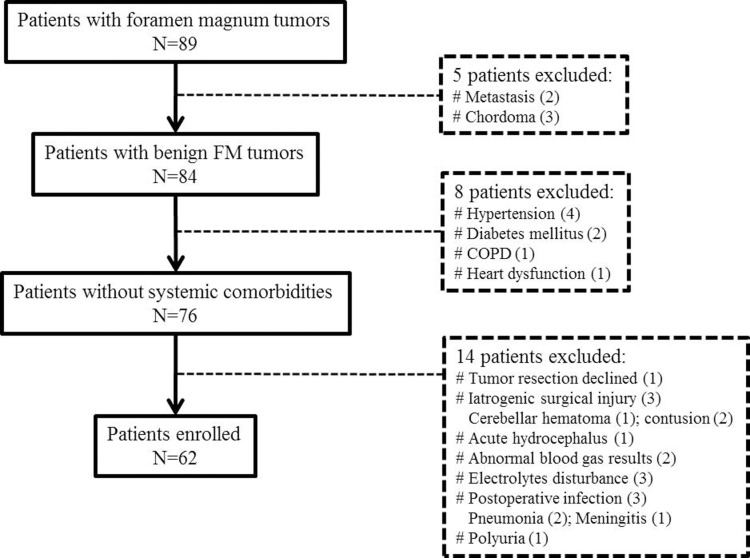
Flowchart of patients enrollment. COPD, chronic obstructive pulmonary disease; FM, foramen magnum.

A total of eligible 62 patients with benign tumors in FM were enrolled in this study. They were treated between January 2010 to January 2015 at the neurosurgical department of West China Hospital, Sichuan University. The surgical resections were all done by the senior neurosurgeon(Yuekang Z). The demographics, radiological data, surgical and histological records, postoperative vertebrobasilar vasospasm were reviewed retrospectively. All records were de-identified and analyzed anonymously. Vertebrobasilar vasospasm was diagnosed by bedside TCD according to the Soustiel’s criteria[17, 18]. BA velocity >70cm/s and BA/EVA ratio >2 were defined as vertebrobasilar vasospasm and BA velocity > 85cm/s, and BA/EVA ratio >3 as severe vasospasm. Once severe vasospasm was confirmed by additional computed tomography angiography (CTA), the anti-vasospastic treatment strategy was initiated immediately. Patients with the following risk factors affecting the value of TCD were excluded: previous chronic diseases of vital organs, postoperative complications such as marked intracerebral/subarachnoid hemorrhage and brain edema/ischemia due to iatrogenic surgical injury, acute hydrocephalus, hypothalamic dysfunction, electrolytes disturbance, pneumonia and meningitis, unstable vital sign and abnormal results in blood gas analysis. The detailed flowchart was shown in Fig 1. This study was approved by Ethics committee of West China Hospital.

Tumor size was defined as the largest diameter of tumor on the preoperative MR imaging. Tumors were classified as anterior/anterolateral, and postolateral according to the location. Lower third clival involvement, extradural extension and vertebral artery(VA) encasement were assessed on enhanced three-dimension MR imaging preoperatively. Surgical information including surgical time, intraoperative bleeding, surgical approach were collected from surgical records. Significant intraoperative bleeding was defined as loss of blood >600ml. The degree of resection was determined by MR imaging within 3 days after operation and divided into three categories: total, subtotal and partial resection. Histological type was noted from pathological record. The vertebrobasilar vasospasm was evaluated 1 day before operation and on postoperative day 1, 3 and 7 by TCD diagnostic system by the senior technician (Shirong Q). The detailed technique was performed as described previously[18].

### Statistical analysis

SPSS 22.0 software (IBM Corp., Armonk, NY, USA) was used for all statistical analyses. Categorical and parametric variables were analyzed using χ^2^ test and unpaired t test for statistics, respectively. Univariate analysis was first performed to detect factors significantly correlated with vasospasm. Then, a multivariate analysis was performed to identify independent risk factors predicting the occurrence of vasospasm in the posterior circulation. Variables with P < 0.1 in the univariate analysis were put into the multivariate analysis model. A backward stepwise method was used and a P value less than 0.05 was regarded as significant.

## Results

Of the 62 patients enrolled in this study, 23 (37.1%) were male. The age ranged from 19 to 71 years with an average of 48.7±12.5 years. The mean tumor size was 28.8±12.0mm (range: 10 to 60mm). 39 (62.9%) tumors were situated anteriorly or anterolaterally while 23 (37.1%) posterolaterally. 16 (25.8%)tumors had VA encasement. Either suboccipital (44, 71%) or far lateral approach (18, 39%) was selected depending on the lesion features and the surgeon’s experience. The overall surgical time was about 5 hours ranging from 3.5 to 7 hours. Total resection was achieved in 55 (88.7%), subtotal resection in 3 (4.8%) and partial resection in 4 (6.5%) patients. Concerning the histologic type, there were 34 (54.8%) meningiomas, 17 (27.4%) neurinomas, 4 (6.5%) neurenteric cysts, other 7 (11.3%) tumors including 4 epidermoids, 2 teratomas and 1 hemangioblastoma.

Based on the diagnostic criteria of TCD, no patient experienced vertebrobasilar vasospasm preoperatively. However, 27 (43.5%) patients were found to suffer from vertebrobasilar vasospasm at a mean time of 3.5 days after operation, of whom 5 (8%) had severe vasospasm. 4 of the five severe vasospastic patients were validated by a followed CTA. The detailed information of the possible risk factors associated with postoperative vasospasm was listed in **Table 1**.

**Table 1:** Baseline data and clinical variables related to postoperative vasospasm.

**Variable**	**Baseline**	**Vasospasm**	**No vasospasm**	**P value**
	(n = 62)	(n = 27)	(n = 35)	
**Age(years)**	48.7±12.5	44.5±12.8	52.0±11.3	0.018
**Male**	23(37.1)	12(44.4)	11(31.4)	0.293
**Tumor Size(mm)**	28.8±12.0	34.0±10.6	24.7±11.6	0.002
**Location**				0.590
**anterolateral**	39(62.9)	18(66.7)	21(60.0)	
**posterior**	23(37.1)	9(33.3)	14(40.0)	
**Clivus involved**	30(48.4)	15(55.6)	15(42.9)	0.443
**Extradural extent**	16(25.8)	8(29.6)	8(22.9)	0.546
**VA encasement**	16(25.8)	12(44.4)	4(11.4)	0.003
**Surgical time(hours)**	4.9±0.9	5.2±0.9	4.7±0.8	0.012
**SIO bleeding**	6(9.7)	4(14.8)	2(5.7)	0.390
**Surgical approach**				0.074
**suboccipital**	44(71)	16(63.3)	28(27.3)	
**far lateral**	18(39)	11(36.7)	7(72.7)	
**Resection degree**				0.675
**total**	55(88.7)	23(85.7)	32(81.8)	
**subtotal**	3(4.8)	2(6.1)	1(9.1)	
**partial**	4(6.5)	2(8.2)	2(9.1)	
**Pathology**				0.850
**meningioma**	34(54.8)	15(55.6)	19(54.3)	
**neurinoma**	17(27.4)	8(29.6)	9(25.7)	
**neurenteric cyst**	4(6.5)	2(7.4)	2(5.7)	
**others**	7(11.3)	2(7.4)	5(14.3)	

VA: vertebral artery; SIO Bleeding: significant intraoperative bleeding

Unilateral statistical analysis (Table 1) showed that younger age (p = 0.018), larger tumors (p = 0.002), VA encasement (p = 0.003), longer surgical time (p = 0.012) were correlated with higher incidence of vasospasm. Far lateral approach had a tendency of higher incidence of vasospasm without statistical significance (p = 0.074). Other factors including gender, location, clival involvement, and significant intraoperative bleeding exerted no significant effect on postoperative vasospasm. In the multivariate analysis (**Table 2**), only age (odds ratio [OR] = 0.936, 95% confidence interval [CI]: 0.888–0.987; P = 0.014) and VA encasement (OR = 8.367, 95% CI: 2.015–34.749; P = 0.003) were identified as independent risk factors which can predict the occurrence of postoperative verterobasilar vasospasm.

**Table 2:** Multivariate analysis of factors affecting postoperative vasospasm.

**Variable**	**Vasospasm**	
	OR(Cl)	P value
Age	0.936(0.888–0.987)	0.014
Size	1.043(0.981–1.109)	0.178
VA encasement	8.367(2.015–34.749)	0.003
Surgical time	0.560(0.160–1.961)	0.364
Surgical approach	0.640(0.164–2.491)	0.640

OR: odd ratio; Cl: confidence interval

## Discussion

To the best of our knowledge, this is the first investigation focusing on vertebrobasilar vasospasm following cranial base tumor resection and on vasospasm in the FM region. Our study showed that the acute vertebrobasilar vasospasm was not an uncommon event with 43.5% of patients diagnosed at a mean time of 3.5 days after FM tumor resection and 8% categorized as severe vasospasm. Age and VA encasement were independently associated with postoperative vasospasm. These results are of clinical importance which can aid in the selection of patients who are at high risk of vertebrobasilar vasospasm and potential brainstem ischemia after FM tumor removal. The early recognition and timely anti-vasospastic treatment of at-risk patients can be life-saving.

Symptomatic vasospasm was a very severe complication with a high mortality and morbidity after brain tumor resection. That 3 (60%) of 5 patients died of ischemic events induced by vasospasm after tumor resection was reported previously[19]. The overall mortality reached 30% in a recent systemic review[5] although Bejjani et al. showed that marked clinical improvement was obtained in most of symptomatic patients under aggressive endovascular intervention[3]. Hence, early detection and the timely management of vasospasm before clinical symptoms is extremely advantageous to improve patients’ surgical outcome. In the present study, no symptomatic vasospasm appeared in 5 patients with severe TCD-detected vertebrobasilar vasospasm after timely “3H” and nimodipine treatment.

Posterior fossa tumors accounted for relatively less symptomatic vasospasm in comparison with anterior/middle fossa lesions after operation[7, 20]. However, nearly all types of tumor in the posterior fossa can be encountered to be associated with cerebral vasospasm[3, 21–24]. Moreover, it was revealed that more than half of patients with acoustic neurinomas presented anterior circulation vasospasm postoperatively[6]. Therefore, postsurgical vasospasm following posterior fossa tumor resection may be underestimated and undiagnosed. It is urgent to assess the real incidence and define the risk factors affecting its occurrence. FM is a region in the posterior fossa where extra- and intracranial VA and basilar artery are located[25, 26]. Tumors in FM usually have a close relationship with vertebrobasilar artery which can be adhered, displaced, distorted or totally encased, and mechanical manipulation of these arteries may run through the whole surgical procedure. Therefore, it is highly representative and of clinical importance to investigate vasospasm of vertebrobasilar artery after tumor removal in FM region.

Many causative factors have been identified responsible for cerebral vasospasm after tumor resection. Alotaibi et al. divided these contributing factors into three categories: preoperative, intraoperative and postoperative[5]. Preoperative factors included tumors located in the sella region with suprasellar extension, tumors narrowing or encasing vessels. Intraoperative factors involved direct injury to or excessive manipulation around vessels, increased cerebrovascular reactivity due to surgery-induced physiological stress. Postoperatively, presence of subarachnoid hemorrhage on CT, complications such as hypothalamic dysfunction, electrolytes disturbance, and meningitis may also induce vasospasm. Other factors including age, tumor size, total operative time, tumor consistency and cerebral metabolic changes were also proposed[6, 27]. Among these factors, the tumor-vessel relationship and blood bleeding into subarachnoid space were regarded as decisive[3, 5]. However, the limitations of the previous literatures in common were lack of statistical analysis or of small sample size.

Our result of younger age significantly related to higher incidence of vertebrobasilar vasospasm was consistent with the findings of previously published studies. Numerous reports indicated the effect of age on the incidence of vasospasm after aneurysmal subarachnoid hemorrhage (aSAH) although the governing factors of vasospasm may be quite different from those after tumor resection. Malinova et al. found that age < 38 years best predicted vasospasm after aSAH in the multivariate analysis[28]. Kale et al equally showed a greater risk of vasospasm in the younger patients with aSAH. Age <50 years was at 5-fold greater risk of vasospasm compared with older patients[29]. Torbey et al. discussed the impact of age on cerebral blood flow velocity and incidence of vasospasm after aSAH, suggesting that older patients had a lower mean flow velocity and a lower incidence of symptomatic vasospasm[30]. Moreover, in a large cohort of 470 consecutive patients with cranial base tumors, the patients developing postoperative vasospasm was usually young with a mean age of 48.5 years[3]. Additionally, an average age of 41 years was identified in a review of cerebral vasospasm after tumor resection[5]. The pathophysiological mechanisms for the decreasing incidence of vasospasm with young age is not well understood. It was plausibly explained by increased vasoconstrictive reactivity of cerebral vessels in younger and decreased response to endothelin in elder patients[31, 32].

Cerebral artery encasement is a common phenomenon in skull base tumors which is associated with a great likelihood of surgical complications and tumor recurrence[33]. However, the relationship of vessel encasement and postoperative vasospasm was seldom studied. It was revealed that there was a statistical difference in the incidence of vessel encasement between patients with or without vasospasm after operation[3], which was in line with our result. Preoperative vessel encasement by tumor can reduce the local cerebral blood flow reserve and require more vessel manipulation intraoperatively to achieve complete tumor resection. The pathophysiological explanation may include direct mechanical trauma to artery, mechanical irritation of the smooth muscle cells or the vasa nervorum, and impaired vasodilatation process[3, 5, 34]. Vertebrobasilar vasospasm occurring at a mean time of 3.5 days after operation in our study indicated that direct mechanical vessel irritation instead of metabolic product of red blood cell may be much more responsible, and indirectly supported that VA encasement contributed to the occurrence of vasospasm in the posterior fossa.

The current study has several limitations. This is a retrospective investigation with innate drawbacks such as selection bias and incomplete data collection from medical chart. We focused on the acute vasospasm within 1 week after operation. Although symptomatic vasospasm may occur as long as 1 month after tumor resection, the average time is approximately 1 week. In addition, CTA was only performed to confirm the presence of severe vasospasm, not done in all patients, so the value of TCD as the sole instrument diagnosing vertebrobasilar vasospasm should be evaluated in further prospective studies.

## Conclusions

Acute vertebrobasilar vasospasm is not uncommon after a FM tumor resection. Age and vessel encasement are the independent risk factors predicting the occurrence of postoperative vasospasm. For patients of younger age and patients with the presence of vertebrobasilar encasement on the preoperative imaging, intensive monitoring by TCD may facilitate early diagnosis and treatment.
